# A Systematic Review of the Advances and New Insights into Copy Number Variations in Plant Genomes

**DOI:** 10.3390/plants14091399

**Published:** 2025-05-06

**Authors:** Saimire Silaiyiman, Jiaxuan Liu, Jiaxin Wu, Lejun Ouyang, Zheng Cao, Chao Shen

**Affiliations:** 1 Guangdong Provincial Key Laboratory for Green Agricultural Production and Intelligent Equipment, College of Biological and Food Engineering, Guangdong University of Petrochemical Technology, Maoming 525000, China; 19990885586@163.com (S.S.); 18375432965@163.com (J.L.); 15077668902@163.com (J.W.); ouyanglejun@gdupt.edu.cn (L.O.); 2 College of Life and Geographic Sciences, Kashi University, Kashi 844000, China; 3 Key Laboratory of Biological Resources and Ecology of Pamirs Plateau in Xinjiang Uygur Autonomous Region, Kashi 844000, China; 4 Maoming Agricultural Science and Technology Extension Center, Maoming 525000, China; caozheng5192@163.com

**Keywords:** copy number variations, agronomic traits, genetic improvement, multi-omics, artificial intelligence

## Abstract

Copy number variations (CNVs), as an important structural variant in genomes, are widely present in plants, affecting their phenotype and adaptability. In recent years, CNV research has not only focused on changes in gene copy numbers but has also been linked to complex mechanisms such as genome rearrangements, transposon activity, and environmental adaptation. The advancement in sequencing technologies has made the detection and analysis of CNVs more efficient, not only revealing their crucial roles in plant disease resistance, adaptability, and growth development, but also demonstrating broad application potential in crop improvement, particularly in selective breeding and genomic selection. By studying CNV changes during the domestication process, researchers have gradually recognized the important role of CNVs in plant domestication and evolution. This article reviews the formation mechanisms of CNVs in plants, methods for their detection, their relationship with plant traits, and their applications in crop improvement. It emphasizes future research directions involving the integration of multi-omics to provide new perspectives on the structure and function of plant genomes.

## 1. Introduction

In recent years, with the rapid development of high-throughput technologies, whole-genome scans have revealed a large number of different forms of sequence polymorphisms at the DNA level. These include single nucleotide polymorphisms (SNPs), small insertions/deletions (InDels), simple sequence repeats (SSRs), variable number of tandem repeats (VNTRs), and CNVs, all of which serve as molecular markers, thereby enriching the content of genomic genetic variation studies [1]. The increased recognition of CNVs in modern genomic studies has ushered in a new phase in the use of molecular markers [1].

**Figure 1:**
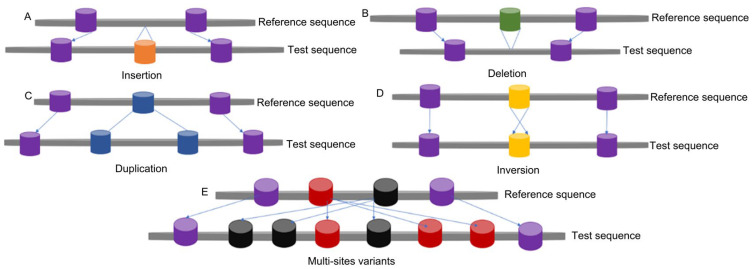
Several structures of copy number variations. Copy number variations (CNVs) refer to submicroscopic chromosomal structural variations ranging in size from 1 kb to several Mb in the presence of a reference genome, when DNA fragments in different individual genomes are compared with the reference genome. This schematic depicts several types of copy number variations in the test genome (lower line) compared with the reference genome, including insertion (**A**), deletion (**B**), duplication (**C**), inversion (**D**), and complex multi-site variations (**E**).

CNVs, as another common type of polymorphism in human, animal, and plant genomes, are changes in the number of copies of a specific DNA sequence in the genome that varies between individuals. Early studies defined CNVs as genetic variations involving DNA segments larger than 1 kb [2], and later this definition was expanded to include DNA segments of approximately 100 bp [3]. Currently, the size of CNVs ranges from 50 bp to several megabases [4,5]. CNVs primarily include insertions, deletions, duplications, inversions, and complex multi-site variants (Figure 1) [3]. CNVs can be classified differently based on their characteristics and impacts. According to the type of variation, they can be categorized into gain-of-copy variations and loss-of-copy variations [6]. Gain-of-copy variations refer to an increase in the number of copies of a particular DNA segment within the genome, often leading to gene dosage effects, which may enhance the expression of associated genes. Loss-of-copy variations refer to the absence of a particular DNA segment within the genome, which can lead to the loss of function of related genes or a reduction in their expression levels. According to their genomic location, CNVs can be classified into intragenic CNVs and intergenic CNVs [7]. Intragenic CNVs occur within the coding regions of genes and directly affect gene function. Intergenic CNVs occur in the non-coding regions between genes and may influence the function of gene regulatory elements. According to their impact range, CNVs can be categorized into small-scale CNVs and large-scale CNVs [8]. Small-scale CNVs typically refer to variations within a few kilobases, while large-scale CNVs involve more extensive genomic rearrangements and may encompass simultaneous variations in multiple genes. Therefore, different CNVs can lead to abnormalities in gene structure and alterations in gene expression [9]. CNVs can be detected in regions without genes as well as in regions containing protein-coding genes or important regulatory elements [10]. CNVs that overlap with genes often destroy their structure and impair their function, affecting expression levels, or may also affect gene regulation through position effects [11,12].

CNVs were initially studied primarily in humans [13,14]. To date, over 50,000 CNVs have been detected in the human genome, accounting for approximately 10% of the entire genome [15]. CNVs are associated with many complex diseases and are frequently used in human disease prevention and clinical diagnosis. For example, having a lower-than-average copy number of *CCL3L1* is significantly associated with increased susceptibility to HIV/AIDS, indicating that *CCL3L1* plays a crucial role in the pathogenesis of HIV/AIDS [16]. Specific genomic regions with CNVs may be associated with certain conditions such as autism, schizophrenia, epilepsy, Parkinson’s disease, or Alzheimer’s disease [17,18,19].

CNVs have also been studied in other animal species. For instance, in cattle, common CNV regions (CNVRs) have provided important genomic information for identifying genes associated with beef quality and meat production efficiency [20]. In the study of the Jeju Korean horse population, functional analysis showed that genes related to olfactory function and neural response were highly expressed in CNVRs [21]. Furthermore, studies in chimpanzees, a mammalian species, have shown that intra-specific CNVs are common in the chimpanzee genome, and a subset of duplication and deletion events may recur both between and within species [22]. Studies have shown that the loss or gain of CNVs can lead to abnormal gene function, restructuring of gene architecture, changes in gene dosage and expression levels, thereby causing phenotypic variations and contributing to the onset and development of certain diseases [23,24,25].

In contrast, CNVs in plants have not been studied so thoroughly. CNVs in the *Arabidopsis thaliana* genome lead to changes in gene content, particularly large deletions or insertions caused by transposable element activity, which to some extent reflect the evolutionary history of the *Arabidopsis* genome [26]. The first crop to undergo CNV detection was corn (maize), where array-based comparative genomic hybridization (CGH) technology was used to detect 3741 CNVRs between the inbred lines B73 and Mo17. Approximately 55% of these CNVs were found to be generated by haplotype-specific tandem duplication events [27]. Genome-wide sequencing of corn has identified numerous candidate gene segments associated with the improvement of corn traits [28]. A total of 2886 CNVRs were detected in the whole rice genome, and the genes located in the CNVs region or overlapping with the CNV region are mostly related to adverse stresses, such as those leading to cell death [29]. In barley, the first systematic CNV map of diploid *Triticeae* species was constructed, revealing that CNV diversity in wild barley is higher than that in cultivated species, suggesting a bottleneck effect during domestication [30]. A key CNV was discovered in cucumber that controls the female flower phenotype, and its formation mechanism was analyzed, laying the foundation for functional gene mining and breeding applications [31]. An efficient CNV detection method suitable for breeding practice was developed in soybean, and the resistance of soybean to cyst nematodes was improved by regulating the copy number of Rhg1, demonstrating the great potential of CNV as a molecular marker in crop improvement [32]. Large-scale CNVs were found in potato, accounting for 219.8 Mb (30.2%) of the entire genome, mainly enriched in gene clusters related to environmental stress response, driving rapid environmental adaptation and evolution by affecting stress-related pathways [33]. A genome-wide CNV variation map was constructed in the *Arabidopsis* population, revealing the distribution pattern of CNVs in natural populations, affecting genes and transposable elements, and participating in population structure, adaptive evolution, and gene expression regulation [34]. By analyzing the copy number variation of the rice Rf4 gene, it was revealed that humans optimize the hybrid breeding strategy of the CMS system by selecting high-copy restorer genes [35]. De novo genome assembly of white clover revealed that CNVs in its cyanogenic genes play an important role in its ability to rapidly adapt to the environment [36]. Further, through population genomics analysis of the invasive plant ragweed (*Ambrosia artemisiifolia*), it was found that CNVs played a key role in achieving rapid and parallel local adaptation in invasive plants [37].

This review summarizes the formation mechanisms of CNVs in plants, detection methods, their relationships with plant traits, and applications in crop improvement. It emphasizes the future research direction of multi-omics integration and provides new insights into the structural and functional studies of plant genomes.

## 2. Mechanisms of CNV Formation

**Figure 2:**
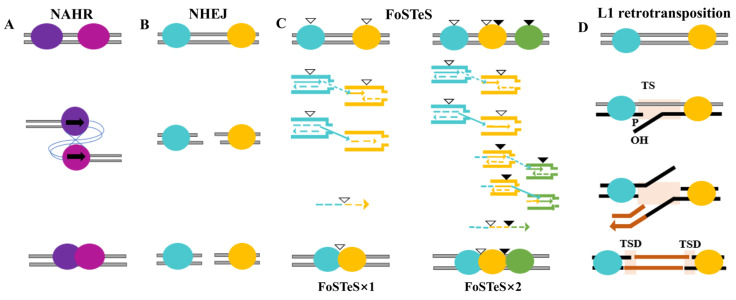
Four major mechanisms of CNV formation. (**A**) Models for non-allelic homologous recombination (NAHR). Non-allelic homologous recombination (NAHR) occurs between highly similar but non-allelic sequences (purple circles), leading to duplication or deletion of intervening genomic regions. Black arrows indicate the direction of homologous recombination events. (**B**) Non-Homologous end joining (NHEJ). Non-homologous end joining (NHEJ) repairs DNA double-strand breaks (yellow circles) without the requirement for extensive sequence homology. Minimal or no homology at the breakpoints can result in small insertions or deletions. (**C**) The fork stalling and template switching (FoSTeS) mechanism involves replication fork stalling (white and black triangles) and template switching events (dashed arrows) during DNA replication. FoSTeS × 1 refers to a single FoSTeS event that leads to simple rearrangements; FoSTeS × 2 refers to two or more FoSTeS events that lead to complex rearrangements (colored circles). Triangles represent short sequences that share microhomology. Each group of triangles (filled or hollow) represents a group of sequences that share the same microhomology with each other. (**D**) L1 retrotransposition. The process involves target site nicking (TS), primer binding (P), and reverse transcription (OH), followed by insertion of the L1 element (brown arrow) and formation of target site duplications (TSDs) flanking the insertion site.

CNVs originate from intrinsic properties of the genome. The main mechanisms for rearrangement in the genome include non-allelic homologous recombination (NAHR), non-homologous end joining (NHEJ), fork stalling and template switching (FoSTeS), and L1-mediated retrotransposition, which are also the causes of most CNVs (Figure 2). Each mechanism reflects a different biological context and leads to different CNV patterns (Figure 2).

### 2.1. Non-Allelic Homologous Recombination (NAHR)

Most CNVs are mainly caused by the NAHR mechanism, that is, crossover recombination occurs between non-allelic homologous DNA sequences with high sequence similarity, resulting in inter-chromosomal, inter-chromatid, and intra-chromosomal structural rearrangements (Figure 2A) [38,39]. In general, segmental duplications (SDs) with sequence homology between 95% and 97% and a length greater than 10 kb or low copy repeats (LCRs) scattered on chromosomes can serve as substrates for NAHR [38,39]. Due to the directional non-overlap of homologous sequences, NAHR can induce duplications, deletions, and inversions of extensive DNA fragments, thereby altering gene copy numbers [3].

### 2.2. Non-Homologous End Joining (NHEJ)

NHEJ is a key DNA double-strand break (DSB) repair mechanism, particularly in response to oxidative damage and ionizing radiation [40]. During the repair process, the DNA ends are processed, often leading to the insertion of a few bases at the junction site (Figure 2B) [41]. Unlike NAHR, NHEJ does not require a homologous DNA template. In contrast, it relies on the nucleotide structure at the breakpoints and is inherently prone to generating duplications and deletions of DNA segments [41]. In addition, CNVs mediated by NHEJ frequently arise near specific sequence motifs associated with DSB formation or DNA bending, such as the TTTAAA motif [42].

### 2.3. The Fork Stalling and Template Switching (FoSTeS)

During DNA replication, FoSTeS occurs when the replication fork stalls, causing the lagging strand to detach from its template. It then transfers and anneals to a nearby replication fork via microhomology at the 3′ end (shared by both the original and invading strands), thereby restarting DNA synthesis and leading to the formation of CNVs. If the new replication fork is located downstream of the original fork, template switching results in the deletion of DNA fragments; if it is upstream, it can cause fragment duplication. Additionally, the orientation of the reattached fragments—whether forward or reverse relative to the original orientation—depends on whether the leading or lagging strand is involved and the direction in which the new replication fork progresses (Figure 2C) [43], and plays a potential role in an increasing number of complex pathological rearrangements [44,45].

### 2.4. L1 Retrotransposition

Long interspersed element-1 (L1), which is approximately 6 kb in length, is the only known active autonomous retrotransposon. It comprises two intact open reading frames: ORF1, encoding an RNA-binding protein, and ORF2, which encodes a protein endowed with both endonuclease and reverse transcriptase activities. L1-mediated retrotransposition is also one of the mechanisms for the formation of CNVs (Figure 2D) [40]. In contrast to NAHR, NHEJ, and FoSTeS/microhomology-mediated break-induced replication (MMBIR), it is a transposition mediated by RNA as a template [46], which starts from the target-primed reverse transcription (TPRT) process [47] and changes other mobile elements in the genome through transduction, such as short interspersed elements (SINEs) and Alu, thereby affecting gene expression [48].

Understanding the mechanisms of these variations is the basis for studying their biological functions, and efficient detection technology is the key to further exploring the impact of CNVs on plant traits and resistance. With the advancement of sequencing technology, we have been able to capture CNVs in plant genomes more accurately and reveal how they work in complex genomes. Next, we will explore how these detection methods can help us identify and quantify CNVs in plants, which will further help us understand the role of copy number in plant growth and development, important phenotypic traits, biotic and abiotic stresses, and domestication, thereby providing new tools and perspectives for crop improvement and gene function research.

## 3. Methods for Detecting CNVs

### 3.1. The Development History of Sequencing Technologies

In 1977, DNA sequencing technology experienced a revolutionary breakthrough. The chemical degradation method (Maxam–Gilbert method) and the dideoxy chain-termination method (Sanger sequencing) enabled systematic determination of DNA sequences, marking a new era in molecular biology research [49]. In the 21st century, second-generation sequencing technologies (also known as high-throughput sequencing) began to emerge, including Roche/454, SOLiD, and SOLEXA sequencing technologies [50,51]. The key feature of second-generation sequencing technologies is their ability to process multiple samples in parallel, enabling faster and more economical genome sequencing [52]. Currently, several commercial platforms use third-generation DNA sequencing technologies, such as Pacific Biosciences (PacBio) Single Molecule Real Time (SMRT) sequencing and Illumina’s TruSeq Synthetic Long-Read technology [53,54]. The representative of fourth-generation sequencing technology is the Oxford Nanopore sequencing platform [55]. These methods have average read lengths between 5 and 15 kilobase pairs (kbp) and can exceed 100,000 base pairs [56]. The development of sequencing technology has greatly promoted the research progress of CNV detection methods [57].

### 3.2. CNV Identification Algorithms and Tools

Traditional CNV detection methods, such as Multiplex Ligation-dependent Probe Amplification (MLPA) and array-based Comparative Genomic Hybridization (aCGH), are considered the gold standard for CNV detection [58]. These methods provide reliable detection results by directly comparing the copy number of the sample to a reference genome. However, they often require complex experimental designs and higher costs, which limit their application in large-scale population studies [58].

There are four main methods for detecting CNVs with NGS data: assembly-based (AS), read-depth (RD), read-pair (RP), and split-read (SR) methods (Table 1) [59,60,61,62,63,64,65,66,67,68]. AS primarily focuses on the representation and behavior of different alleles (inherited information from both parents) at specific genomic positions [59]. RD refers to the number of times a specific genomic position is covered by sequencing reads generated by the sequencer [60,61,62,63]. RP refers to read pairs generated by paired-end sequencing, consisting of two short reads that are oriented in opposite directions and originate from the two ends of the same DNA molecule [64,65]. SR refers to short DNA sequences generated by high-throughput sequencing technologies, such as those from the Illumina platform. These short reads typically range from tens to hundreds of base pairs in length and are among the most commonly used types of sequencing data [66,67,68]. Each method described above has its own strengths and limitations. As data volumes increase, the Combined Approach (CA) method emerges. CA utilizes a step-by-step process to integrate data from multiple sources, capitalizing on the unique strengths of each tool involved [69,70,71,72]. Each method has its own advantages and disadvantages, and combining their strengths can lead to better detection results [73].

Popular computer languages used for the development of software/tools for predicting CNV are mainly Python 3.8, C++14, and R 4.1.0. For each of these languages, the existing tools will be described in this review.

Tools developed in Python: Hecaton is a novel computational workflow tailored for plants, which integrates calls from various state-of-the-art algorithms using machine learning methods. Several state-of-the-art tools incorrectly represent dispersed duplications as overlapping deletions and tandem duplications, whereas Hecaton can correctly detect dispersed duplications [74]. ifCNV combines artificial intelligence techniques, using two Isolation Forest algorithms and a comprehensive scoring method, to accurately detect CNVs in various samples. This approach improves the accuracy and reliability of CNV identification, providing a new tool for plant genomics research [75]. HBOS-CNV is a newly proposed method whose core is the use of a new statistical approach, specifically a histogram-based method. By conducting in-depth analysis of the data, this method can effectively identify copy number variations in the genome [76]. CNVpytor is a Python library designed for detecting and visualizing CNVs. It is tailored to handle data from cancer genomes and other complex samples, especially datasets containing repetitive sequences or polyploid genomes [77]. The Magnolya algorithm uses a Poisson mixture model to estimate the copy numbers of contigs assembled from sequencing data. This algorithm does not require mapping reads to a reference genome but instead detects CNVs de novo through co-assembly [59]. SCSilicon can efficiently generate silicon-based DNA sequencing reads for single cells with minimal manual intervention. SCSilicon can automatically create a series of genomic abnormalities, including SNPs, Indels, and CNVs [78].

Tools developed in C++: Control-FREEC is primarily used for detecting CNVs and purity from high-throughput sequencing data. It is particularly suited for cancer genomics research, as tumor samples often contain complex copy number alterations and may be affected by contamination from normal cells, requiring correction of sequencing data [79]. CNVnator is one of the most popular tools for CNV/CNA discovery and analysis. It identifies CNVs based on variations in read depth from sequencing data. By performing statistical analysis on sequencing data, CNVnator can efficiently detect copy number variations in the genome, particularly excelling at detecting short repetitive sequences [61]. CNVer implements an ambiguous mapping strategy that uses all good mappings for each mate pair, resulting in increased sensitivity for repeated and duplicated regions [69]. It is worth noting that CNVeM is able to distinguish genomic regions with only 0.1% difference, thus achieving high-resolution CNV boundary prediction [80].

Tools developed in R: BIC-Seq2 is an upgraded version of BIC-Seq (Bayesian Integer Count Sequencing) that uses Bayesian statistical methods to infer copy number states. It is not only suitable for cancer samples but can also be used for other types of samples, such as those in developmental biology or genetics research [60]. ExomeDepth is a tool designed to detect CNVs from exome sequencing data. It is primarily used to discover copy number variations in cancer samples or other disease samples, especially from Whole Exome Sequencing (WES) data. By utilizing the depth of sequencing reads, ExomeDepth identifies increases or decreases in copy number. This tool has been applied in studies to identify CNVs affecting gene content in barley [81]. The svpluscnv package is a multifunctional toolbox for integrating and interpreting multiple orthogonal datasets, including copy number variation (CNV) segmentation profiles and sequencing-based structural variation (SV) calls. This package implements analysis and visualization tools [82]. JointSLM (Joint Segmentation and Likelihood Model) can simultaneously analyze data from multiple samples, which is particularly useful for identifying common copy number variations in populations [83]. SCYN can efficiently detect and infer copy numbers from single-cell DNA sequencing data [84].

Command-line tools developed in Java: GATK (Genome Analysis Toolkit) is a widely used suite of tools developed by the Broad Institute. It helps researchers perform variant detection, gene expression analysis, copy number variation analysis, and more in genomics research [85]. Allelic variations in soybean germplasm were detected using the GATK toolkit [86]. cnvHiTSeq is a tool that can process standard BAM file format input data and is applicable to various types of sequencing experiments, such as Whole Genome Sequencing (WGS) and Whole Exome Sequencing (WES) [87].

**Table 1:** Copy number variation detection tools.

Software	Language	Name	Year	References
PEMer (-)	Python/Perl	RP	2009	[64]
BreakDancer (Version 1.4.5)	Perl/C++	RP	2009	[65]
SegSeq (-)	MatLab	RD	2009	[88]
Pindel (Version 0.2.5b9)	C++	SR	2009	[68]
mrFAST (Version 2.6.1)	C	RD	2009	[89]
CNV-seq (Version 0.9.7)	Perl/R	RD	2009	[90]
RDXplorer (Version 3.2)	Python/R	RD	2009	[91]
SV Detect (Version 1.4)	Perl	SR	2010	[71]
rSW-seq (-)	C	RD	2010	[92]
cnD (-)	D	SR	2010	[93]
CNVer (-)	C++	CA	2010	[69]
GenomeSTRiP (Version 2.0)	Java/R	CA	2011	[70]
BIC-Seq (Version 0.2.4)	Perl/R	RD	2011	[60]
CNVnator (Version 0.4.1)	C++; Perl	RD	2011	[61]
ReadDepth (Version 0.9.8.1)	R	RD	2011	[62]
JointSLM (-)	R	RD	2011	[83]
PRISM (Version 1.1.6)	C	SR	2012	[66]
SVseq2 (Version 2.2)	C++	SR	2012	[67]
ERDS (Version 1.1)	C	RD	2012	[94]
DELLY (Version 1.1.3)	C++/R	CA	2012	[72]
GASVPro (Version SCR_005259)	C++	CA	2012	[95]
Control-FREEC (Version 11.6)	C++	RD	2012	[79]
CnvHiTSeq (Version 0.1.2)	Java	CA	2012	[87]
Cn.MOPS (Version 1.38.0)	R	RD	2012	[63]
Magnolya (Version 0.15)	Python	AS	2012	[59]
Clever-sv (-)	C++	CA	2013	[96]
SoftSearch (Version SCR_006683)	Perl	CA	2013	[97]
CNVeM (Version 0.710)	C	RD	2013	[80]
CNVrd2 (Version 3.21)	R	RD	2014	[98]
Gindel (Version 0.8)	C++	CA	2014	[99]
PSCC (-)	Perl	CA	2014	[100]
LUMPY (Version 0.3.1)	C; C++; Python; Shell	CA	2014	[101]
Hydra-Multi (Version 0.5.4)	C++	CA	2015	[102]
CNVcaller (Version 1.0)	Python	CA	2017	[103]
GATK4 (Version 4.3.0.0)	Java	CA	2018	[104]
Hecaton (-)	Python	RD	2019	[74]
ExomeDepth (Version 1.1.16)	R	RD	2020	[81]
CONY (-)	R	CA	2020	[105]
inCNV (Version 2.2.0)	Python	RD	2020	[106]
CNVpytor (Version 1.2.2)	Python	CA	2021	[77]
Svpluscnv (-)	R	CA	2021	[82]
SCYN (-)	R	CA	2021	[84]
SCSilicon (-)	Python	CA	2022	[78]

The selection of an optimal tool for CNV detection largely depends on the specific requirements of the study, including the data type, the complexity of the CNVs, and the desired sensitivity. Among the tools, LUMPY [101] and GATK4 [104] stand out for their ability to integrate multiple detection methods, such as RD, RP, and SR, making them highly versatile for detecting both small and large structural variations, particularly in cancer genomics and large-scale genomic studies. These tools are highly regarded for their robustness and accuracy in identifying complex structural variants. On the other hand, CNVpytor [77] and ExomeDepth [81] are more specialized for exome sequencing (WES) data, excelling in detecting small CNVs with high sensitivity. These tools are particularly beneficial for targeted genetic studies and clinical applications where accurate identification of small-scale variations is critical. For single-cell genomic analysis, tools like SCSilicon [78] and CNVrd2 [97] are tailored to handle the challenges posed by high heterogeneity and sparse data, enabling precise CNV detection in individual cells. Overall, the choice of tool should be guided by the specific research context, with GATK4 [104] and LUMPY [101] being ideal for large-scale and cancer-related studies, while ExomeDepth [81] and CNVpytor [77] are better suited for clinical or exome-based research.

## 4. Recent Advances of CNVs in Plant Genomes

**Figure 3:**
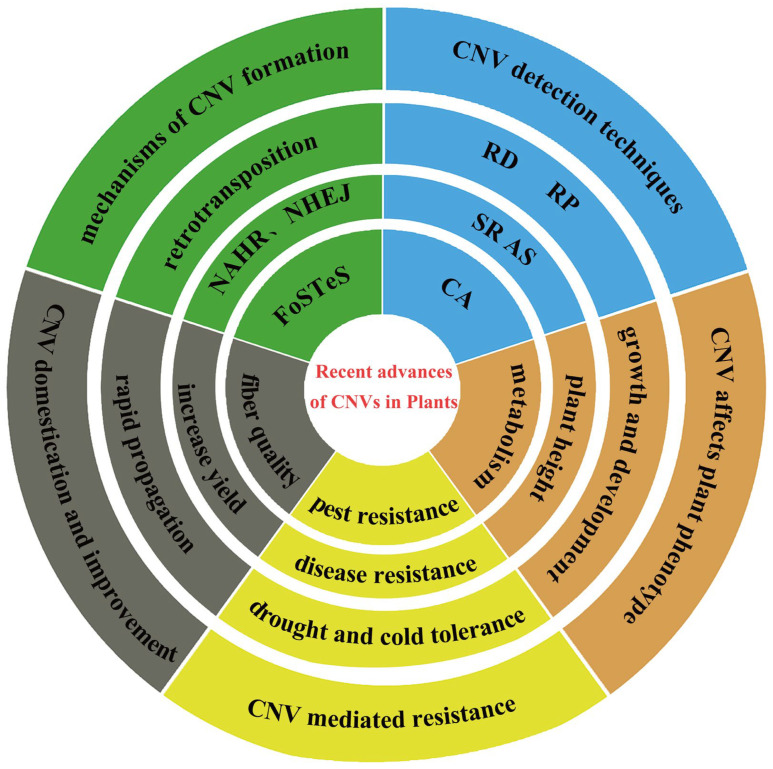
Recent advances in copy number variations. NAHR: Non-Allelic Homologous Recombination; NHEJ: Non-Homologous End-Joining; FoSTeS: Fork Stalling and Template Switching. RD: Read-depth; RP: Read-pair; SR: Split-read; AS: Assembly-based; CA: Combined Approach.

CNVs are not only common in wild species but also frequently occur in cultivated crops (Table 2). The frequency and patterns of CNVs can vary among different plant species and varieties. CNVs can affect gene dosage, thereby influencing gene expression levels and leading to phenotypic changes. Some CNVs are associated with plant stress resistance (such as disease resistance and drought tolerance), which helps plants survive under adverse environmental conditions. CNVs can also impact crop yield and quality. Using high-throughput sequencing technologies and bioinformatics tools, researchers can effectively detect and analyze these variations, providing crucial information for plant breeding and functional genomics studies (Figure 3).

### 4.1. CNVs Affect the Phenotype of Plants

An increasing number of studies have shown that CNVs are widely present in plant genomes. In plant genomes, the presence of large-scale CNVs affects characteristics such as plant height, growth and development, and metabolic processes [1]. For example, differences in flowering time among wheat varieties are caused by CNVs in the genes *Vrn-A1* and *Ppd-B1* [122]. In corn, a CNV occurs at the qγ27 locus, and duplication at the 27 kDa γ-zein locus (qγ27) is crucial for the conversion of soft endosperm to hard endosperm in Quality Protein Maize (QPM) [123]. Liu et al. (2020) reported a CNV involved in rice architecture by regulating tiller number and leaf angle. It was found that *OsMTD1* not only influences tiller number and leaf angle but also suppresses the transcription of pri-miR156f in the CNV region. This CNV functions through dosage and positional effects on *OsMTD1* and pri-miR156f [124]. By performing quantitative trait loci (QTL) mapping in a recombinant inbred line (RIL) population of 460 lines, a QTL for trailing growth and branch length was identified. Within this QTL, a CNV region was characterized by increased copy numbers of gibberellin 2-oxidase 8A/B, which encode gibberellin 2-oxidase 8. The increase in the copy number of these genes reduced trailing growth and branch length during the domestication of soybeans [125]. More than 700 inter-specific copy number variation regions have been identified in grapes, affecting over 2000 candidate genes that may lead to phenotypic differences between varieties [126]. Cucumber has a unique genetic system for female sex expression, which is determined by a dominant and dosage-dependent female (F) locus based on copy number variation [110]. A total of 4715 CNVs were identified in 24 lotus accessions, including 448 duplications and 4267 deletions, and their population structure was further analyzed, laying the foundation for subsequent exploration of the impact of population CNVs on phenotypes [111]. CNVs affect 30.2% of the potato genome, with nearly 30% of genes being at least partially deleted or duplicated, revealing the highly heterogeneous nature of the potato genome [33]. Association analysis of leaf development and disease resistance traits related to 103 maize lines was conducted using SNPs and CNVs. The study found that CNVs make a significant contribution to the variation of the analyzed phenotypes and provide complementary information to SNPs [127]. These variations not only enrich phenotypic diversity in plants but also offer significant potential for improving crop yield and quality.

### 4.2. CNVs Enhance Plant Tolerance to Adverse Environments

CNVs play a key role in plant responses to environmental challenges and are closely related to resistance to stresses such as drought, high temperature, pests, and diseases [1]. By selecting varieties with specific CNVs, it is possible to enhance crop adaptability to unfavorable environmental conditions [128]. The CNV duplication of the *ZmLOX5* gene has a quantitative contribution to maize insect pest defense, and its introduction into high-performance but insect-susceptible crop varieties can enhance plant resistance to insect pests and tolerance to abiotic stresses [121]. In soybean, CNVs in *rhg1* (*GmSNAP18*) were found to contribute to resistance only in lines derived from PI88788 and ‘Cloud’, and at least 5.6 pi88788-type *rhg1* copies were required to obtain Soybean Cyst Nematode (SCN) resistance, regardless of the *Rhg4* (*GmSHMT08*) haplotype. However, when the *GmSNAP18* copy number was below 5.6, a ‘Peking’-type *GmSHMT08* haplotype was required to ensure SCN resistance. This suggests a novel epistatic mechanism between *GmSNAP18* and *GmSHMT08* involving a minimum requirement for copy number [86]. By resequencing the resistant barley variety “Nure” and comparing it with the sensitive variety “Morex”, the results showed that the presence of CNV proximal to the locus of the resistant variety “Nure” increased the frost resistance of barley [129]. CNVs are associated with nucleotide-binding leucine-rich repeat (NB-LRR) genes and receptor-like kinase (RLK) genes, which are involved in plant defense mechanisms [130]. Several disease resistance genes enriched for specific biological functions related to cell death, protein phosphorylation, and defense responses were found within the CNV regions of rice [29]. Studies on the genetic mechanism of copy number variation of resistance genes in Cucurbitaceae revealed that R gene loci are often lost in different Cucurbitaceae species [131]. Genome sequencing confirmed that five NBS-LRR genes were missing in the An subgenome and three were missing in the Cn subgenome of rapeseed, which may reflect different selections for disease resistance in rapeseed [132]. CNVs affect gene expression and defense mechanisms, helping plants better adapt to adversity, thereby improving crop resistance and yield quality [1]. This genetic-level improvement will help improve the stability and sustainability of agricultural production in the context of climate change and environmental degradation.

### 4.3. CNVs Accelerate the Domestication of Plants

The domestication of plants is a complex historical event involving the transition from wild ancestors to cultivated varieties. During this process, CNVs, as an important form of genetic variation, have a significant impact on plant adaptability, stress resistance, and economic traits. CNVs are a ubiquitous source of genetic variation in domesticated taxa. Early studies of CNVs in domesticated species used very few samples [133]. For example, a genome-wide comparison of two varieties in rice found 641 CNVs ranging in size from 1.1 kb to 180.7 kb [134]. Analysis of two maize inbred lines revealed 400 genomic regions showing duplications and widespread presence/absence variations (PAVs) affecting over 700 genes [28]. Array comparative genomic hybridization (CGH) was used to compare gene content and copy number variation in 19 different maize inbred lines and 14 maize wild ancestor teosinte genotypes. Compared with B73, 479 genes had higher copy numbers in some genotypes, and 3410 genes had lower copy numbers or were absent in at least one genotype. This suggests that these variants predate domestication and that no strong selection has acted on them [135]. CNV at the Grain Length on Chromosome 7 (GL7) locus contributes to grain size diversity in rice [136]. CNVs can undergo a transient phase of CNV fixation during domestication; for example, in the African rice *Oryza glaberrima*, LOF in *PROG1*, which controls the transition from prostrate to erect growth, was caused by a gene loss relative to the ancestral locus [137]. If the domestication phenotype is caused by separate but independent mutations, CNVs can be observed in domestication genes within domesticated species. For example, loss of seed shattering is a key domestication trait observed in cereal crops [138]. Both SNP and deletion alleles were observed at the sorghum *sh1* locus, which resulted in loss of the seed shattering trait. The deletion CNV of *sh1* remained polymorphic in sorghum; further comparison revealed that it was selected in parallel during the domestication of sorghum, rice, and corn [139]. These studies highlight parallel evolution and multiple origins of domesticated species, all elucidated through the study of CNV mutations, suggesting that CNV promoted the domestication selection of plants.

### 4.4. CNVs Promote Genetic Improvement in Plants

With global climate change and population growth, improving crop yields and their ability to withstand biotic and abiotic stresses has become an important goal of agricultural research. Significant copy number variations have been found in certain gene regions related to drought tolerance in rice [139]. Introducing these characteristics into main cultivated varieties through traditional breeding or molecular breeding methods can improve rice yield and stress resistance [140]. CNVs related to disease resistance found in wheat can significantly improve wheat’s disease resistance [141]. There is a CNV at the high-density planting adaptability (HPDA-D12) locus on chromosome D12 of the cotton mutant AiSheng98 (AS98), and its association with the expression of *GhDREB1B* leads to the phenotype of the AS98 mutant. Overexpression of *GhDREB1B* significantly reduced plant height and branch length and reduced branch angle. Finely regulating the expression of *GhDREB1B* may be a feasible engineering strategy to improve cotton plant architecture to adapt to high-density planting [113]. A study of Resistance Genes Analogues (RGAs) in eight *Brassica napus* lines found that CNVs were more likely to appear in clustered resistance genes (RGAs) than in single resistance genes [118]. In addition, 112 disease resistance genes are associated with quantitative trait loci (QTL) for blackleg resistance, 25 of which are affected by copy number variations. This finding can advance the breeding of rapeseed lines [118]. In soybean cyst nematode (SCN)-resistant varieties, copy number changes of a 31-kb repeat encoding multiple gene products were observed among different haplotypes at the *Rhg1* locus [142]. The cloning of *Rhg1* is the first observation that a plant disease resistance locus can be composed of a multi-gene cluster CNV formed by the concatenation of atypical resistance genes; in SCN-susceptible varieties, there is one copy of a 31 kb fragment in each haploid genome. SCN resistance was found to be associated with increased expression of CNV-related genes [143]. Some disease resistance genes account for a large proportion of genes in CNV regions and are significantly enriched in resistance gene models [144,145]. For example, 876 CNV regions were identified in apple, covering 3.5% of the apple genome, and genes related to apple disease resistance were enriched [146]. In peanuts and legumes, the R gene undergoes extensive copy number variation [147]. High copy number of resistance genes in plants is expected to be advantageous as it will provide better resistance to pathogens [131]; low copy number may be due to less challenge from pathogens [148]. These studies reveal that CNVs can promote genetic diversification and the evolution of new resistance genes. By understanding and exploiting these variations, breeders can develop higher-yielding, better-quality crop varieties to meet the world’s growing food demand.

## 5. Future Prospects

With the advancement of genomic technology, CNV detection methods have become increasingly diverse, from basic cytogenetic methods to molecular-based technologies such as array comparative genomic hybridization (aCGH) to high-throughput sequencing (NGS). The application of these technologies has made the identification and analysis of CNVs more efficient and accurate, thus providing a powerful tool for sustainable agriculture, but it also faces a variety of technical challenges.

### 5.1. Enhanced Detection Sensitivity and Accuracy

The complexity of sequencing data is a major challenge in CNV research. Due to the diversity and complexity of plant genomes, especially polyploid plants, the repetitive sequences and structural variations in their genomes make it difficult to accurately detect CNVs. Different sequencing platforms and technologies (such as second-generation sequencing and third-generation sequencing) have differences in data quality and resolution capabilities, which will affect the identification and quantification of CNVs [149]. The quality of genome assembly directly affects the detection effect of CNVs. In plant genome research, splicing errors during the assembly process may lead to the appearance of false-positive CNVs. In addition, inaccurate genome annotation will also affect the functional interpretation of CNVs [150]. At present, there are still challenges in data sharing and standardization in CNV research. Different laboratories and research groups use different sequencing platforms and analysis processes, which reduces the comparability of data. In order to promote the progress of CNV research, it is necessary to establish unified data standards and sharing platforms [151]. Current tools often struggle with detecting low-frequency CNVs, particularly in heterogeneous samples such as cancer or single-cell populations. Future research is likely to focus on developing more refined algorithms that can accurately detect CNVs at lower frequencies, improve the detection of subclonal CNVs in tumors, and handle the complexities inherent in rare or complex structural variations.

### 5.2. Integrating Multi-Omics Data to Study CNV

Multi-omics is a powerful tool for understanding biological complexity and accelerating the research process of plant copy number variation. The main advantage of multi-omics is that it provides a holistic understanding of biological systems. By integrating data from different omics, the relationship between various biological processes can be revealed. For example, in plant research, copy number variation of the genome may affect the transcriptome and proteome, thereby affecting the phenotype and adaptability of plants [151]. Multi-omics approaches can improve plant breeding efficiency to improve the nutritional value of wild species, crop yields and resistance to biotic and abiotic stresses, thereby achieving sustainable food security [152]. In plant breeding, multi-omics can help identify genes and regulatory mechanisms associated with specific traits. By analyzing the multi-omics data of different plant varieties, genotypes with excellent traits can be selected, thereby accelerating the breeding process and improving crop yield and stress resistance [153]. The future will likely see more comprehensive multi-omics approaches that combine CNVs with transcriptomic, epigenomic, and proteomic data. Multi-omics will continue to play an important role in various fields of biology, helping us gain a deeper understanding of the nature of life.

### 5.3. Artificial Intelligence Revolutionizes CNV Detection

The application of artificial intelligence technology in CNV has begun to show its potential. Artificial intelligence technology, especially machine learning and deep learning, can accelerate the data processing and analysis process, reduce research costs, and make CNV analysis more efficient. For example, researchers used Hecaton, a new computational workflow, to detect CNVs in plant genomes, combining genomic data and transcriptome data, significantly improving the detection ability of CNVs [74]. Using deep learning algorithms, CNVs can be effectively detected from simulated and known copy number variations. This method outperforms traditional coverage estimation methods and can more accurately identify the type and location of variants. For example, dudeML performs quite well in detecting copy number variations, especially in samples with low coverage, using statistics that are easy to derive from samples. These tools are not computationally intensive and can be used in many data sets to detect duplications and deletions for a variety of purposes [154]. AI can also play an important role in gene editing. By accurately detecting key sites and designing guide RNA, AI can help breeders achieve more efficient gene editing and directly improve genes related to CNVs [155]. The application of this technology enables researchers to extract valuable information from complex data, thereby accelerating the process of plant breeding and genetic improvement.

The application of CNV research in plant breeding will be further deepened. The following are some development directions: (1) Interdisciplinary and cross-species cooperation: Combining multi-omics knowledge, such as genomics, transcriptomics, epigenetics, phenotyping, ecology, etc., by integrating CNV data from different crops, revealing their common genetic mechanisms and laws of adaptive evolution, we can gain a more comprehensive understanding of the biological mechanisms of CNVs and their role in plant breeding. (2) Precision design breeding: With the development of genomic selection and machine learning technology, more accurate AI models are being developed based on the characteristics of plant CNVs to improve the efficiency and accuracy of CNV detection and analysis, helping breeders select suitable parents based on specific CNV information and improve breeding efficiency. Breeding in the future will be more personalized and can be optimized for specific environments and needs. (3) Sustainable development: With the global attention to sustainable agriculture, CNV research will provide important support for the development of stress-resistant and high-yield crops, and help meet the challenges brought by climate change.

## 6. Conclusions

CNV research in plants has broad prospects. The future of CNV research is poised for significant advancements with the integration of cutting-edge technologies, including long-read sequencing, multi-omics approaches, and AI-driven analysis. For example, combined with modern genomics and artificial intelligence technology, CNV will not only provide a new perspective for breeding but also provide an important tool for improving crop productivity and adaptability. Despite some challenges, with the continuous advancement of technology, CNV research will play an increasingly important role in future plant breeding, bring new impetus to the development of modern agriculture, and provide new possibilities for achieving sustainable agriculture and improving food security.

## Figures and Tables

**Table 2:** Summary of plant copy number variation studies.

Category	Publication Year	No. of Samples	CNV Detection Tools	References
Glycine max	2019	106	GATK, SAMTools	[86]
Potato	2019	47	GATK	[107]
*Arabidopsis thaliana*	2020	1060	CNVnator	[34]
Rice	2020	93	CNVnator, Delly, CtgRefCNV	[108]
hexaploid wheat	2020	16	GMAP	[109]
Barley	2020	397	ExomeDepth	[81]
Cucumber	2020	9	FISH qPCR	[110]
Lotus	2020	24	Delly, Manta	[111]
Tomato	2020	100	SVCollector	[112]
Cotton	2020	2464	GATK	[113]
Poppy	2020	10	CNVnator	[114]
Cotton	2021	1961	CNVcaller	[115]
Chili Pepper	2021	2	Illumina HiSeq X-ten, NovaSeq 6000	[116]
Sorghum	2022	400	GATK	[117]
*Brassica napus*	2022	8	CNVnator	[118]
Grape	2023	82	2−DDCt method, QuantStudio	[119]
Apple	2023	346	SpeedSeq, Lumpy, CNVnator	[120]
Zea mays	2024	6	ddPCR	[121]

## Data Availability

The data presented in this study are available on request from the corresponding author.
